# Synthesis and Characterization of an Alkali-Activated Binder from Blast Furnace Slag and Marble Waste

**DOI:** 10.3390/ma17215248

**Published:** 2024-10-28

**Authors:** Gülden Çagın Ulubeyli, Recep Artır

**Affiliations:** 1 Department of Civil Engineering, Zonguldak Bulent Ecevit University, 67100 Zonguldak, Turkey; 2 Department of Metallurgical and Materials Engineering, Marmara University, 34722 Istanbul, Turkey; recep.artir@marmara.edu.tr

**Keywords:** alkali-activated slag, marble waste, workability properties, mechanical characteristic, drying shrinkage, microstructural analysis

## Abstract

This study reports an alkali-activated binder including blast furnace slag (BFS) together with marble waste (MW). Cement is an industrial product that emits a significant amount of CO_2_ during its production and incurs high energy costs. MW is generated during the extraction, cutting, and processing of marble in production facilities, where dust mixes with water to form a settling sludge. This sludge is an environmentally harmful waste that must be disposed of in accordance with legal regulations. In this study, a substantial amount of MW, a by-product with considerable environmental and economic impacts worldwide, was utilized in the production of a binder through the alkaline activation of BFS. In doing this, different experimental parameters were tested to obtain the best binder samples according to workability and mechanical properties. Then, some experiments such as drying shrinkage determination, strength testing, and microstructure analyses were fulfilled through samples with the best values. The findings supported the improvement of the rapid-setting property of BFS by means of the addition of MW. MW reduced the time-dependent drying shrinkage values of BFS by 55%, especially in slag alkaline activation systems with a low or moderate alkali activator content. The substitution of MW (≤50%) in BFS increased flexural and compressive strengths (4.5 and 61.7 MPa), while a reference sample contained BFS only. Although the use of MW did not create a new phase, it contributed to a C-S-H bonding structure during the alkali activation of BFS in a microstructure analysis.

## 1. Introduction

To minimize ordinary Portland cement (OPC) concrete, one of the most suggested substitutes in the literature is alkali-activated binders. This type of binder requires a green production technique that has low carbon emissions and forces the use of industrial waste by-products [1]. In general, aluminosilicate materials include industrial by-products and agricultural waste. However, fly ash (FA) and blast furnace slag (BFS) have been widely and thoroughly studied so far as the most prevalent source materials [1,2]. In theoretical binder synthesis mechanisms, alkali materials can be used to activate silica, calcium oxide, and alumina [3]. Therefore, new sustainable products based on alkali activation that reuse CaO- and SiO_2_-rich products have gained importance. Raw materials used in the process include waste and secondary products from different industrial processes.

In this study, the binding properties of two materials activated with alkaline activators, namely BFS and waste marble dust, were investigated. BFS can react with and be effectively activated by sufficient catalysts such as alkaline solutions [4]. A number of past research efforts have examined the mechanical properties and workability as well as the volumetric stability behaviors of binder materials produced by activating BFS with various alkaline activator solutions [5,6,7,8,9,10,11,12]. The durability properties of binder samples through the use of the alkali activation of BFS under the influence of sulfate [13], acid [14], high CO_2_ (carbonation) [15], and high temperature [16,17] effects have also been determined. Considering all these studies, two main disadvantages have been identified in the binder production process of BFS with an alkaline activation system. These are (i) micro-cracks following rapid setting and (ii) excessive drying shrinkage [18,19,20]. The rapid setting issue [21,22] and excessive drying shrinkage as well as the inability to provide volumetric stability [23,24] in the alkali activation of BFS have led to challenges in commercializing the product and have limited its large-scale production. This has necessitated the examination of the contribution of different additives and materials to the activation process in order to eliminate these disadvantages in the alkaline activation process of BFS and expand the industrial usage limits of the binder. Therefore, in this study, waste marble dust was utilized to address the aforementioned disadvantages in the alkali activation of BFS, thereby improving the workability, drying shrinkage, and mechanical characteristics of the binder.

Given that the addition of waste marble powder in alkali activation has been investigated, only a few examples of research have been found. There is only one previous study for a one-component (unary) activation of marble waste (MW) [25]. It implemented the activation of MW sludge with various air, moisture, and water contents and measured the best compressive strength values between 38 and 45 MPa after air-curing for 28 days. However, the workability characteristics of fresh samples were not investigated. In the current research, in addition to producing a binder material through the unary constituent activation of MW, the effect of MW on the alkali activation of BFS was also examined. Hence, it was anticipated that MW could be recycled to yield a new binder, transforming it into a sustainable product while ameliorating the drawbacks of BFS in activation work. The workability properties of waste marble powder, which directly affects the mechanical and long-term characteristics of the resulting binder, were also examined.

There are very few examples of research where MW and BFS have been activated as binary binders. In these studies, workability, strength, and microstructure analyses have generally been conducted using alkali-activating marble powder and slag with different activator solutions [26,27]. In one of these studies, MW led to a low compressive strength for sodium-carbonate-activated slag mortars, but it had an agreeable value. In a microstructure analysis, MW was found to have no hydration activity [26]. In another study, the findings demonstrated both the adjusted fluidity of slurry through MW and an improved strength of cementitious materials by a low-alkali liquid modulus. The activated MW cementitious material revealed Ca(OH)_2_ and Ca_6_(Si_2_O_7_)(OH)_6_ structures in the geopolymer unit [27]. In this study [27], the effects of MW particle size and quantity on the workability properties of various alkali-activated mixtures incorporating slag and metakaolin were also examined. The workability results showed that in samples where slag and MW were combined, an increase in the MW content led to a decrease in the slurry’s fluidity rate and an increase in its viscosity. This effect was attributed to the high water demand of MW. However, this observation was not in agreement with the findings of the current study. Moreover, in the present research, the workability property was measured not only in terms of fluidity but also by determining the setting time. In the current study, the workability, strength, and microstructure properties of alkali-activated BFS, including MW, and its volumetric stability, such as drying shrinkage, were evaluated. Thus, the possibility of converting alkali-activated slag into a common industrial product increased.

In the related literature, there are many studies that use MW with other waste/by-products [28,29,30]. However, considering (i) its unary alkali activation process and (ii) its binary alkali activation process with BFS, only a few past research examples exist. Moreover, to better comprehend the activation of MW regarding its strength, microstructural, and durability properties, the alkali activation processes of certain minerals (namely, calcite, dolomite, and limestone), which have a chemical structure similar to marble, are still under investigation.

For example, Ortega-Zavala et al. [31] studied alkaline cements via limestone activated by water glass and sodium hydroxide. For materials activated through Na_2_O of 7%, the biggest annual strength was 24 ± 2.28 MPa for the material with Ms: 1.5 (Ms is the ratio of SiO_2_/Na_2_O within alkali solutions). They showed that limestone was an inexpensive and common raw material and that the binders could be used instead of Portland cement. In [32], calcite and dolomite minerals were employed in different proportions from 0 to 100%. As a result, 100% calcite content provided 9.3 MPa strength after 28 days for Ms: 1.2.

There are some studies in which limestone and BFS were involved in the binary and ternary component activation [33,34]. Rakhimova et al. [34] determined that limestone content of up to 60% increased the compressive strength at around 50%. Limestone also led to a better physical structure and compressive strength value, providing a greater density by keeping the water content steady [34]. Given some other research efforts, the limestone content of 20% [35] and 30% [36] in a mixture of BFS and fly ash improved its strength values. Generally speaking, limestone did not create new phases, but increased the water content somewhat [36]. Overall, limestone increased the strength of the activated BFS. According to the related studies, the substitution of limestone, which has a property similar to the chemical composition of MW, in BFS enhanced its workability properties [34,35]. These studies indicated that the improvement in the workability could be attributed to the filling of micro-cracks in limestone. In practice, this required less water and allowed for better particle packaging. Consequently, the availability of the additional water can facilitate the lubrication of particles and increase the slump flow [34,35]. In another study, it was found that the addition of limestone of 20% to the slag/fly ash mixture reduced the plastic viscosity of samples [36]. In the current study, unlike these previous studies, the workability and durability properties of the binder type produced with the unary and binary alkaline activation of MW were investigated. The effect of MW on some drawbacks or disadvantageous properties such as the workability loss and the loss of the volumetric stability was also examined while BFS was activated in the binary alkali activation samples. The purpose of this study was to produce different binders that may be employed in place of ordinary cement through the activation of MW and BFS in the binary mixtures. To achieve this, some properties of the binder obtained after an alkali activation process as a unary and binary binder of MW and BFS were analyzed. These properties were identified through some analyses, such as the workability, strength, durability, and microstructure, and were compared to those of the reference sample obtained by a unary binder of BFS. Consequently, the paper highlights how waste materials can be employed as a binder in place of ordinary cement. Such an effort will likely lead to low energy costs and greenhouse emissions, thereby contributing to the emergence of a sustainable material.

## 2. Experimental Studies

### 2.1. Raw Materials

**Figure 1:**
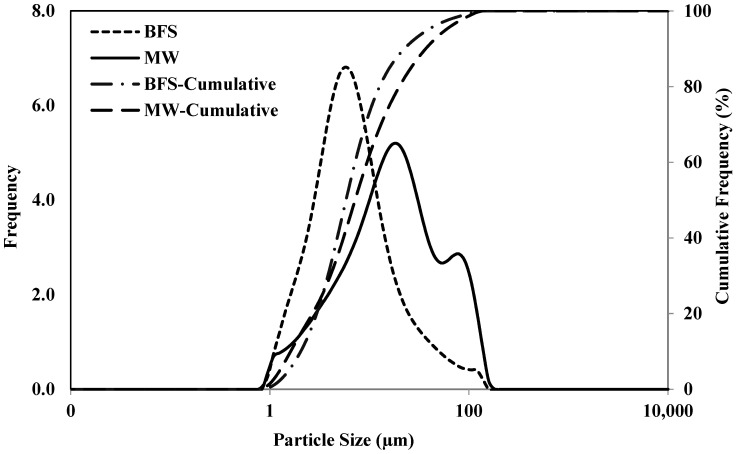
Particle size distribution for raw materials.

Two raw materials (i.e., BFS and MW) and alkali activators (sodium hydroxide and liquid sodium silicate) were utilized. As an additive material, lime was used. The activated BFS pastes were taken as control samples. Before the experiments, material characterization analyses were carried out for raw materials. Also, the fineness and specific gravity were obtained by the Blaine (BET, Quantachrome Autosorb IQ) and pycnometer (Ultrapycnometer 1200e) tests. The basicity coefficient (Kb) of BFS was 0.73, while its hydraulic module was 1.18. The particle size distribution was measured through Mastersizer 2000 (Malvern Instruments, Worcestershire, UK). Both BFS and MW had similar results from 1 to 100 µm (Figure 1). BFS was finer when compared to MW.

The chemical analyses for BFS and MW (Table 1) were performed via XRF (Panalytical Epsilon 5).

**Figure 2:**
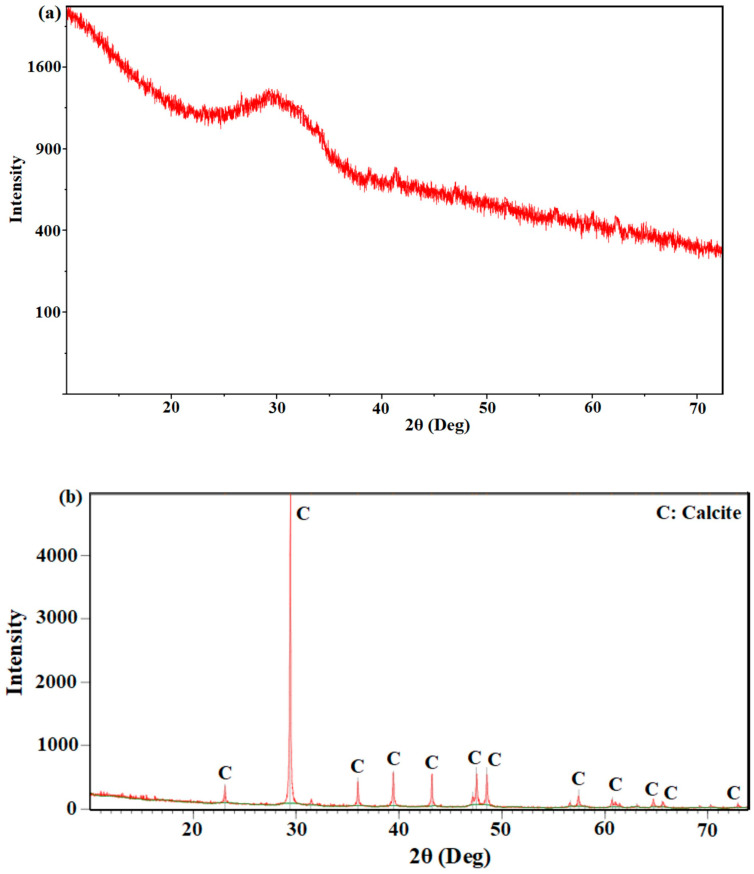
XRD results: (**a**) BFS; (**b**) MW.

The mineralogical ingredients of BFS and MW were measured (Figure 2) through XRD (Panalytical Empyrean with Cu Kα radiation, 45 kV and 40 mA). BFS showed an amorphous texture. Thus, only minor crystal phases were available. In previous studies, very similar results were obtained. For instance, BFS had a hump at 25–35° for 2θ [16,37,38]. MW was acquired from marble mud including water and marble dust. Therefore, similar to past studies, calcite was effective in MW [25,31,39]. As given in Table 1, the XRF and XRD analyses pointed out the existence of the same component, namely calcium carbonate.

**Figure 3:**
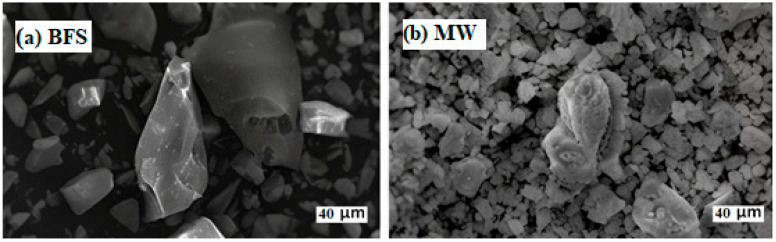
SEM results: (**a**) BFS; (**b**) MW.

For the morphologies of BFS and MW, SEM (Quanta FEG 450) was used. These images indicated the non-uniform and cornered particles for BFS, while the micrographs showed a wide dispersion for the MW particles (Figure 3).

### 2.2. Preparation of Samples

The binary pastes were activated by sodium hydroxide and sodium silicate. Here, two important parameters (that is, “n” and “Ms”) had an impact on the properties of paste samples. From these parameters, “n” showed the total of Na_2_O%, while “Ms” indicated the result of SiO_2_/Na_2_O. Three different “n” ratios (2, 4, and 6) and “Ms” ratios (1.2, 1.5, and 1.8) were utilized to select the most appropriate activation parameters. As these parameters were the proportional expressions, they could be viewed as the indicators of the variation in the activator dosage within samples. Conducting some experiments for each value within this range would considerably increase the number of samples produced. Therefore, our objective was to experimentally evaluate these two ratios (i.e., “n” and “Ms”) across as wide a range as feasible to determine the optimum sample production, and to assess how variations in these parameters influenced the properties of samples. The NaOH solution was prepared a day in advance with the distilled water. The liquid solution, composed of sodium hydroxide, sodium silicate, and water, was simultaneously added to the dried pastes just before the mixing process. The ratio of sodium silicate to sodium hydroxide in the activator solution varied depending on the activator parameters, specifically the ratios “n” and “Ms.” The water-to-binder volume (w/b) was selected as 0.40 and 0.45 according to the preliminary results. After the flow diameter of each mixture sample was measured using the flow table device (diameter: 254 mm) and the setting times were determined with the full automatic Vicat apparatus (branded Vicamatic-2), the samples were placed into molds. The prismatic samples (40 × 40 × 160 mm) were produced for the mechanical experiments and were air-cured throughout a day under laboratory conditions. Then, they were held in two curing conditions (65 ± 3 °C in an oven and steam for 4 h). After curing, they were labeled and put in a dryer and wrapped with a stretch film to maintain their current moisture contents. The designs of samples according to these experimental parameters of the binary mixing samples are given in Table 2.

**Figure 4:**
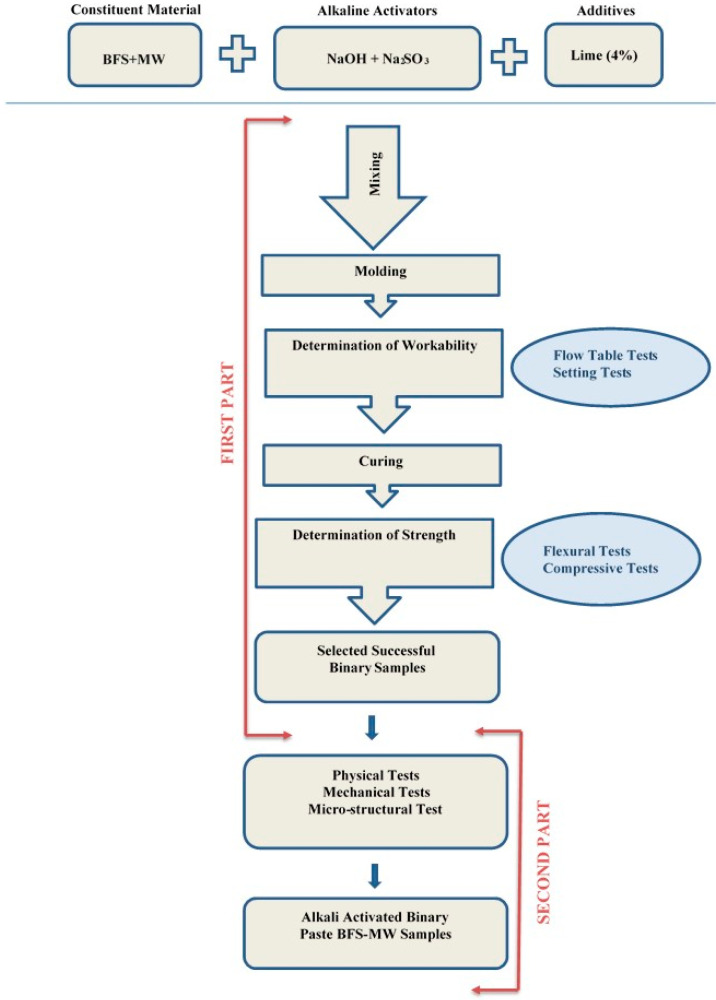
The flow chart of experimental parts.

The experiments were performed in two parts (Figure 4). In the first part, a test sample with the greatest strength and workability (for ease of production of samples during the mixing, molding, and curing stages and the ability to harden in a certain time period) was identified by testing different experimental parameters. Thus, the best samples under the defined experimental conditions were determined. At the first stage, the criterion for selecting the successful samples for the binary binders was defined, since the top three samples with the highest compressive and flexural strength values were separately evaluated. Accordingly, the samples were independently assessed for the compressive and flexural strengths. In the second part, the early and advanced strength properties, the volumetric stability behavior and the microstructure characteristics of the best paste sample were determined and checked against the corresponding control specimen.

The binder paste samples were prepared in two different sizes: a 40 × 40 × 160 mm prism for the mechanical tests and a 25 × 25 × 285 mm prism for the volumetric stability experiments. A flexural/compression frame (capacity: 2000/15 kN), designed and manufactured by the Simulation-Based Engineering Laboratory (SBEL) in Madison, Wisconsin USA, was used for the flexural and compressive strength tests. The drying shrinkage test setup consisted of a length measurement frame equipped with a comparator device. The height change of the mortar bars was measured using a comparator device, with a precision of 0.001 × 12.7 mm. For the alkali-activated paste samples, the materials were processed in a mixer. This mixer had two speeds (140 rpm and 285 rpm). The process was performed at 140 rpm for 1 min, and liquids were supplemented. It was carried out at 140 rpm and 285 rpm, each for 1 min. The alkali-activated samples were placed in molds and were cured in the laboratory throughout a day. After demolding, the samples were held under two different curing conditions at 65 ± 3 °C in an oven and steam.

The sample codes were determined according to the substitution rate of MW in BFS. In the “3BFS-1MW” sample, there was 75% BFS and 25% MW by mass, while in the “2BFS-2MW” sample, there was 50% BFS and 50% MW by mass. Similarly, the “1BFS-3MW” sample contained 25% BFS and 75% MW by mass.

## 3. Results and Discussion

### 3.1. First Part

#### 3.1.1. Workability

##### Flow Table Test

**Figure 5:**
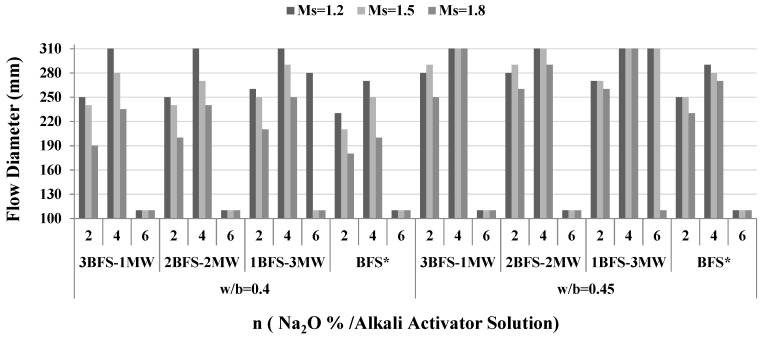
Variations in flow diameter. BFS*—reference sample.

This test was performed using ASTM C 1437-15 [40]. Figure 5 illustrates how the flow diameter varied with different parameters. For the BFS/MW specimens, a rise in the water/binder rate from 0.4 to 0.45 raised the flow diameter. The greater the MW substituted in the BFS-MW specimens, the greater the flow diameters for both w/b ratios. In terms of the Na_2_O-activated BFS-MW specimens, a larger “w/b” led to consistency liquefaction. However, in terms of the BFS-MW binder specimens, an increase in the MW ratio resulted in a considerably longer phase-changing duration from liquid to solid while “n” was 6. The samples with 1BFS-3MW kept their own plastic consistency at large “n” values for 1–2 h. Wang et al. [27] pointed out that MW enhanced the fluid phase for slurry. In this study, it was proposed that an increase in the fluidity could be attributed to the weak adsorption between the liquid lamina on the alkali solution and the marble powder particles. Thus, the marble powder indirectly acted as a “lubricant” in the liquid and contributed to an increase in the fluidity [26,27]. This is consistent with the current findings that the marble in the slag reduced the viscosity and improved the workability.

##### Setting Time Test

**Figure 6:**
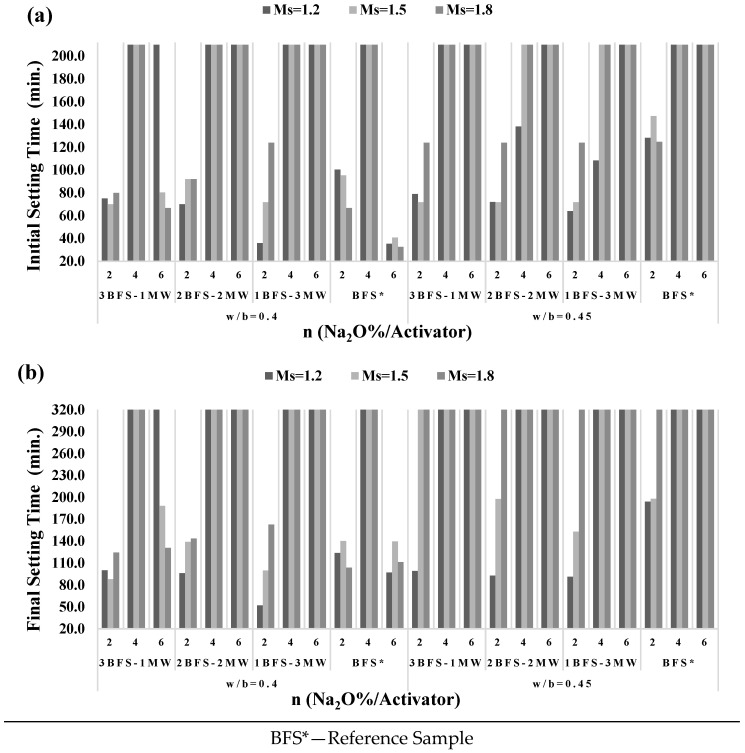
(**a**) Initial and (**b**) final setting duration in BFS-MW specimens.

Figure 6 shows some variations in different setting times of specimens. A rise in the w/b ratio or the MW substitution in the BFS-MW specimens caused a larger setting time. Given the substitution rates of MW in BFS, there was no setting while n = 4 and Ms = 1.8. For 1BFS-3MW, in the case of n = 6 and Ms = 1.5–1.8, the specimens were not set at the end of two days. The changes in the setting duration are illustrated in Figure 6.

The addition of MW to BFS improved the workability in BFS*. The setting time for BFS* from the plastic to solid phases was not long for great amounts of alkaline solutions (Figure 6). MW enlarged the setting duration about two times for big “n” rates and maintained its plastic state in the long run when compared to a reference sample. For BFS-MW, the more the MW, the better the workability. However, “Short Period Difference” was similar to the setting duration while n = 2. A rise in the MW ratio reduced the setting duration, considering small “n” values.

The substitution of limestone with peer chemical characteristics of MW in BFS enhanced the workability because of the fact that the micro-cracks of limestone, which needs little water, were filled. Thus, much water may wash the particles and provide more flowability [34,35]. In another study, limestone (≤20%) in a BFS-fly ash sample decreased the plastic viscosity [36]. In conclusion, the findings of past studies seem to be in harmony with those of the present study on the improvement of the workability in the BFS-MW specimens.

#### 3.1.2. Compressive and Flexural Strengths 

**Figure 7:**
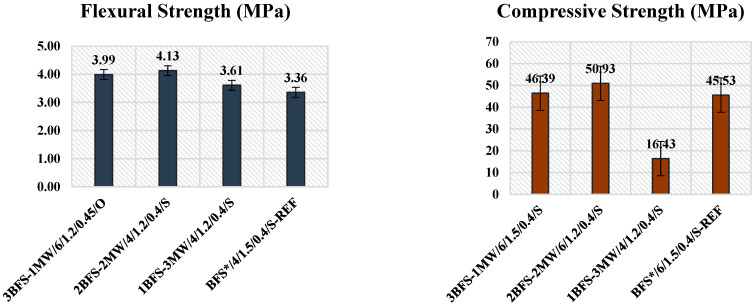
The chosen strengths for the BFS-MW mixtures.

In the binary activated system, MW was substituted with BFS for three masses. Figure 7 illustrates the compressive and flexural strengths in the binary mixtures. The greatest values were those of the 2BFS-2MW mixture. Hence, these values for the binary mixtures were compared to these compositions giving the chosen strengths.

The highest flexural strength for the BFS-MW specimens was that of “2BFS-2MW/4/1.2/0.4/Steam”. It was obtained under the steam curing conditions, while w/b was 0.4. An increase in the amount of MW enhanced the flexural strength of the BFS* specimens for the steam-cured samples. Cappola et al. [25] showed that the marble sludge cured in air through an alkali activation process improved the flexural strength when checked against MW cured in water or moisture. In the current study, the existence of MW provided a higher flexural strength by steam curing. In the binary mixtures, this type of strength was very similar for the specimens that had the chosen value. It means that a rise in w/b from 0.4 to 0.45 affected the strength to a negligible level.

“2BFS-2MW/6/1.2/0.4/Steam” had the greatest compressive strength for the BFS-MW binary specimens. It was found under the steam curing conditions, while w/b was 0.4. While w/b rose from 0.40 to 0.45, the compressive strengths usually declined. There was a negligible gap for this type of strengths for two curing conditions of the binary mixtures. The addition of MW enhanced the strength of the BFS* specimens. Overall, this type of strength improved together with the activator for two curing conditions and for two w/b values.

Two types of strengths for the BFS-MW specimens were found to be better when compared to those of the reference specimens. The “2BFS-2MW/4/1.2/0.4/Steam” specimen with an MW content of 50% had the best flexural strength, and the “2BFS-2MW/6/1.2/0.4/Steam” sample with an MW content of 50% had the best compressive strength. When the target rate of increase was 23% for the flexural strength, the amount of increase in the compressive one was 12%. This means that the growing rate of the flexural strength was about two times greater when compared to that for the compressive one. Despite the fact that an MW content of 75% raised the flexural value, there was a decline of about 64% in the compressive value when BFS* was considered. Given the microstructural analyses in Section 3.2.3, the binary mixture with a C-S-H bond that improved the strength did not lead to a new phase. However, the MW content improved the strength through a “filler effect”. Thus, an increase in the MW content seems to improve the flexural strength rather than the compressive strength.

Considering the specimens with the chosen values of two types of strengths of a week for the binary mixtures, the Ms values were 1.2 in the case of different “n” values. For the flexural strengths, “n” was 4, while it was 6 for the compressive strengths. The finding exposed the improvement of the compressive strengths through an increase in activators. However, a rise in “n” adversely affected the workability issues. Hence, “n” was taken up to 6.

Limestone, which is similar to marble in terms of the chemical characteristics, improved the strength of the activated BFS [34]. It (≤60%) also had a positive impact on the compressive strength at around 50% when checked against the reference specimen. In other studies, it was substituted 20% [36] and 30% [35] within a BFS/fly ash paste and enhanced the strength values. Generally speaking, it did not form new phases, but increased the water content somewhat [35]. It also enhanced the compressive strength, supplying a greater density for a compounded specimen without an increased water content [34].

#### 3.1.3. Selection and Determination of Binary Samples for Workability and Strength

After the workability and strength tests, one sample was separately determined for two types of strengths of the binary mixtures. Table 3 gives some parameters for the chosen specimens. The samples with the highest strength values and with the non-problematic workability were selected.

### 3.2. Second Part

#### 3.2.1. Drying Shrinkage

**Figure 8:**
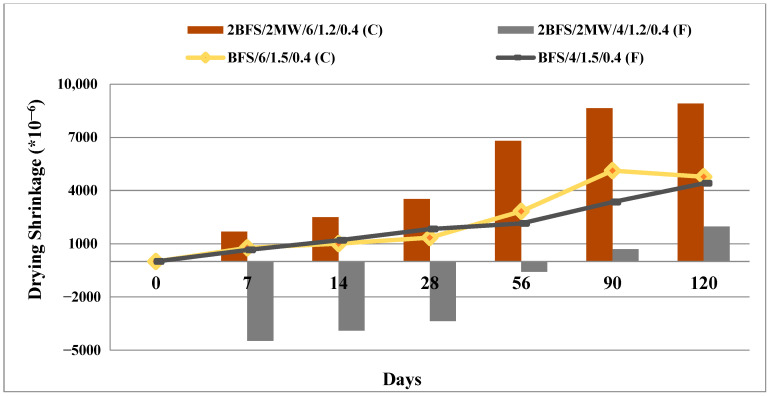
Drying shrinkage results for the samples chosen at the first part.

This experiment was performed on the prismatic samples (25 × 25 × 285 mm) according to ASTM C 596 [41]. Figure 8 illustrates the results for the samples chosen at the first part.

When the binary mixtures had an activator rate of 4, an enlargement rather than shrinkage was detected up to 90 days under the curing conditions. If this rate was 6, the shrinkage of the binary mixtures was considerably greater than the reference specimens after 4 months. Thus, the more the “n” rate, the more the shrinkage.

Although many studies exist about the activated BFS, its drawbacks (namely, micro-cracks [21,22] and drying shrinkage [23,24]) were reported. It has been observed that MW reduced the time-dependent drying shrinkage values of BFS by 55%, especially in the slag alkaline activation systems where “n” was 2 or 4. Given the findings of the present study, MW contributed to the protection of the workability (by prolonging the setting time of the slag) and the volume stability in the alkaline activation system of the slag.

#### 3.2.2. Compressive and Flexural Strength Experiments

**Figure 9:**
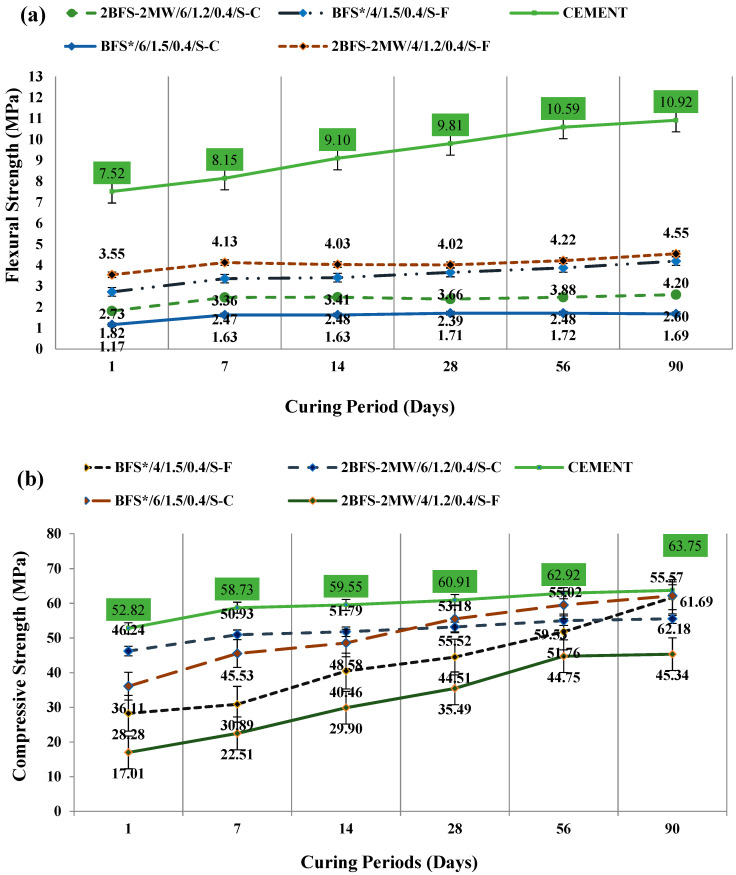
(**a**) Flexural and (**b**) compressive strengths for specimens cured under steam.

Figure 9 illustrates a comparison of the selected flexural and compressive strengths of specimens as of 1, 7, 14, 28, 56, and 90 days under the steam curing conditions.

Given the binary mixtures where BFS and MW had the same ratios (i.e., 50%), the flexural strength slightly increased when compared to the BFS reference samples (BFS*/6/1.5/0.4-C and BFS*/4/1.5/0.4/S-F) in the case of “n” = 4 and “n” = 6. However, the compressive strength was reduced by 15% in the case of “n” = 6, when checked against the BFS*/6/1.2/0.4/S-C reference samples. The greatest values of two types of strengths of the MW specimens were found while “Ms” was set to 1.2. As the ratio of MW increased to 50% in the case of 2BFS-2MW/4/1.2/0.4/S, the corresponding flexural value rose when compared to that of the reference specimens. Considering the same sample, there was an approximately 22% increase observed between the early and late compressive strengths during 28 and 90 days. However, for the samples with an increased alkali activator ratio to “6” (“n” = 6), specifically 2BFS-2MW/6/1.2/0.4/S, only about a 10% increase was noted between the early and late compressive strengths during 28 and 90 days. This indicates that, as the alkali activator solution rate within the sample increased, the rising trend of these compressive values decreased. This finding is in conflict with a previous study in which the MW samples had a rate of 40% within the stone dust under 75 °C and exhibited a slight improvement for the compressive strengths with a more alkali activator solution concentration from 5M to 10M [42]. In fact, the difference may arise from the variations in the primary materials, where MW was substituted, and from the differences in the content of the alkali activator solutions.

#### 3.2.3. XRD, FTIR and Microstructural Analysis 

##### XRD Analysis of BFS-MW

**Figure 10:**
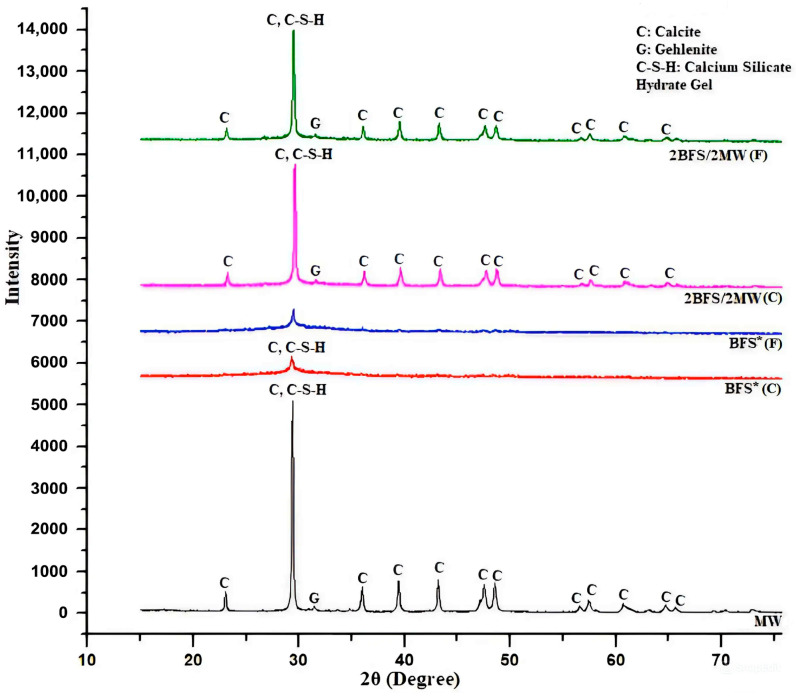
XRD result for the chosen binary specimen.

Figure 10 illustrates the findings of the activated binary mixtures among the chosen high-performance specimens in accordance with the compressive (2BFS-2MW (C)) and flexural strengths (2BFS-2MW (F)).

The peaks of the binary mixtures were identical to those in a one-component alkaline activated system labeled as the MW-Unary Binder. There was no crystal system at 65 ± 3 °C under steam curing conditions. The calcite formation was a significant and dense one. The other small formations were available as thermonatrite (Na_2_CO_3_·H_2_O) and gehlenite (Ca_2_Al [AlSiO_7_]). Considering the XRD outcomes, there was no new phase. The finding was very similar to those of past studies. For example, the mixtures including BFS and limestone neither interacted in a chemical manner nor created a phase [34]. Also, there was no crystalline structure in the activated mixtures that contained the fly ash, BFS, and limestone [35]. MW did not chemically react with BFS, but physically forms a greater density through closing the micro-cracks of BFS [34,35]. Limestone decreased the pore capacity of a BFS/fly ash composition, leading to a rise in the strength [36]. This increased both types of strengths in this study, and could be due to the limestone content and its great packing impact [34].

As shown in Figure 10, the C-S-H peaks and calcite overlapped each other in the XRD results. MW may help form a C-S-H bond, since the rate of Ca to Si rose due to MW. This, in turn, increased the acidic level and reduced the polymerization process when checked against the alkali systems that had a large Si rate [43].

##### FTIR Results for Binary Mixtures

**Figure 11:**
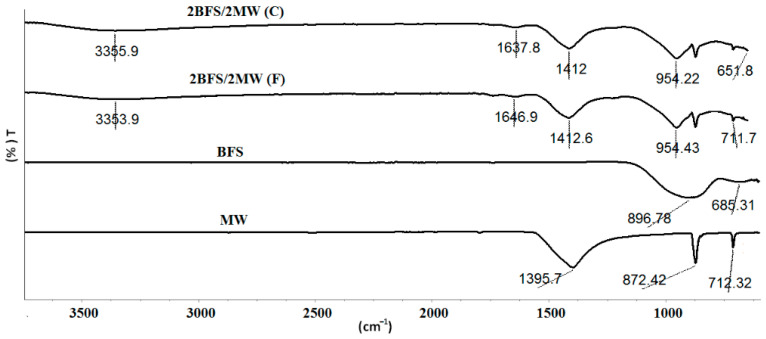
FT-IR result for a chosen binary specimen.

Table 4 gives the FT-IR outcomes for the activated mixtures of the chosen high-performance binary mixtures in accordance with the compressive (2BFS-2MW (C)) and flexural strengths (2BFS-2MW (F)). The FT-IR spectra of both samples are shown in Figure 11.

The Si-O-Si asymmetric stretching vibration peaks within the 850–1200 cm^−1^ range for the raw BFS indicated the presence of an amorphous silicate bond structure [27]. A broad peak at 896.5 cm^−1^ was detected, which denoted the Si-O-Si asymmetric stretching vibration. Since the raw MW was primarily composed of CaCO_3_, the carbonate group vibration peaks were observed at 712.33, 872.28, and 1395.77 cm^−1^ [25,31,45,46]. Also, the spectrum at 1795.30 cm^−1^ for the raw MW suggested the presence of an anhydride carboxyl group [25].

For both samples of the binary alkali activation of BFS and MW, the characteristic carbonate group vibration peaks appeared at 711 and 872 cm^−1^, reflecting the carbonate components that did not react during the alkali activation. However, a strong Si-O silicate bond stretching vibration at 954 cm^−1^ was noted in both alkali-activated samples, which was different from those of the raw BFS and MW [38,43,44]. This peak was the most significant indicator of a SiO_4_ tetrahedral structure that contributed to the cementitious properties provided by the C-S-H bond. The O-H stretching vibrations were also identified at the 3353–3355 cm^−1^ band in both samples, representing the structural water within the C-S-H bond [38,39].

All IR peaks were identical to those of the unary constituent structures for MW and BFS. As a sign of the C-S-H bond at the 950 cm^−1^ band for the alkali-activated BFS, the Si-O band slid to the 954 cm^−1^ band for the binary mixtures. This was confirmed via the XRD outcomes in the previous section (Figure 10). In the case of the binary mixtures, XRD did not show any new phase. At approximately 975–978 cm^−1^ in the systems that had a high SiO_2_ content, an absorption peak in the FTIR slid to the smaller wavelengths (950–955 cm^−1^), since the rate of CaO increased. Here, these peaks of the binary mixtures were detected in the wavelength of about 955 cm^−1^.

##### SEM-EDS Results for Binary Mixtures

**Figure 12:**
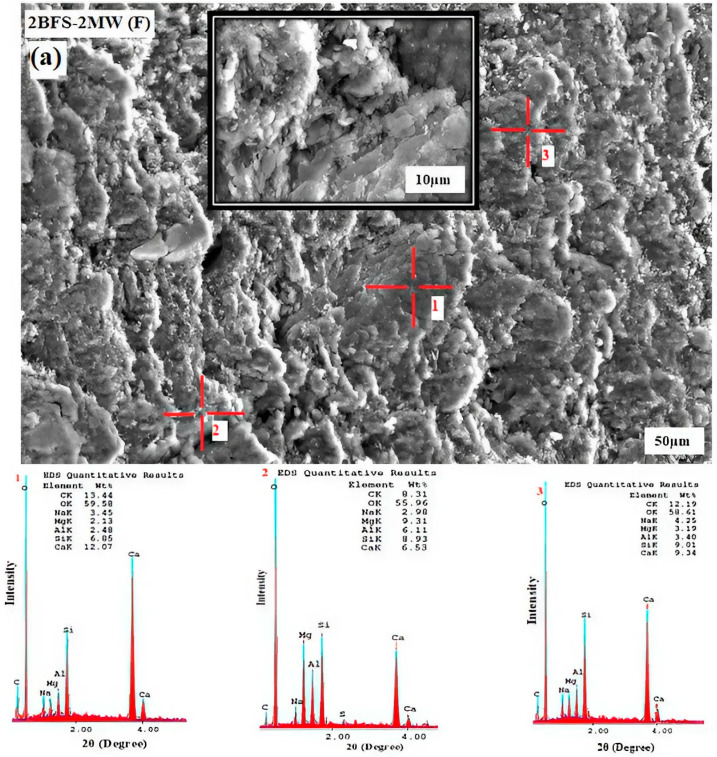

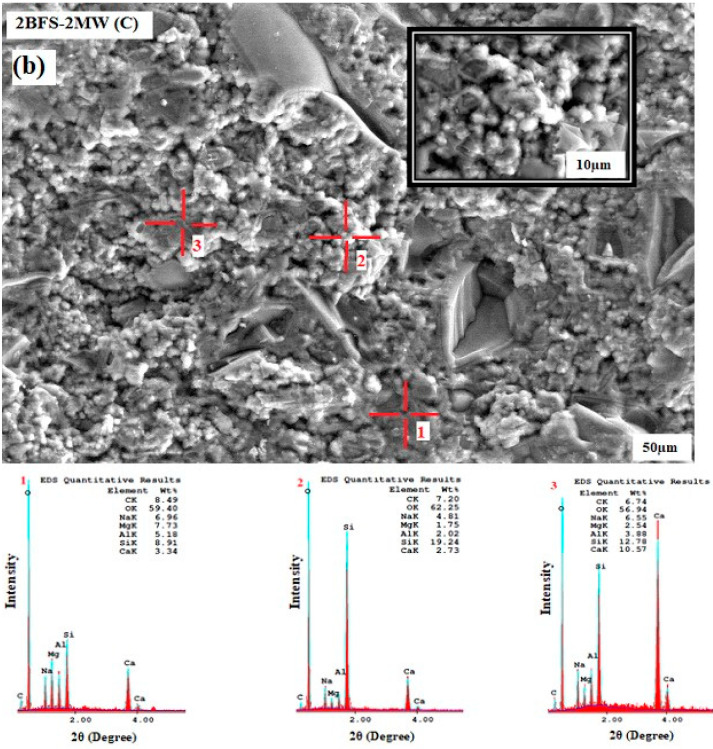
(**a**). SEM-EDS images for the chosen 2BFS-2MW (F) sample. (**b**). SEM-EDS illustrations for the chosen 2BFS-2MW (C) sample.

Figure 12a,b, respectively, show the SEM-EDS outcomes for the chosen high-performance binary mixtures in accordance with the flexural (2BFS-2MW (F)) and compressive strengths (2BFS-2MW (C)).

Figure 12a illustrates the 2BFS-2MW (F) composition with the chosen outcomes after the flexural strength experiment. “Regions 1 and 3” that had a high rate of Ca/Si may indicate a reaction. Considering past studies, sodium carbonate can activate a paste including limestone and BFS, and the Ca/Si rate for a C-S-H bond structure of an end reaction product was higher than the rate of other activated mixtures. This is because of an excessive calcite structure within a mixture [34]. Looking at Figure 12a, the excessive Si ions in “region 2” may be based on how much intense sodium silicate is under non-reaction.

As illustrated in Figure 12b, 2BFS-2MW (C) had the chosen values for the compressive strength. Here, the darker areas without spaces (i.e., region 3) may point out a reaction. Given these areas, the rate of Ca/Si was greater than that of the light particles (region 1 and 2), which were likely related to the raw materials and activators under non-reaction or partial reaction. Similar to this study, Han et al. [47] highlighted that the combined nano-CaCO_3_ particles at a 6% rate in the slag induced a filler effect. This led to the formation of the denser and more homogeneous regions in the SEM images, particularly in the areas where the hydrated gel form developed.

## 4. Conclusions

The production operation of the activated binders has become a leading investigation area because of their nearly non-existent carbon emissions and relatively inexpensive energy expenses. Thus, the current study employed two by-products and/or waste in the industrial manufacturing operations. Given these advantages, it can contribute to the production of sustainable construction materials. The outcomes of the tests for the unary, binary, and ternary samples can be highlighted as follows.

- The more MW in the binary mixtures, the more the flow diameter and the setting duration. In other words, MW may improve the workability of BFS. With a great “n” rate, the duration for the transformation between liquid and solid enlarged.
- In the binary mixtures that had the greatest values of both types of strengths, MW amounts were identical. A substitution of 50% for MW gave the best result. Hence, the substitution rate for MW in BFS can be increased to 50%, thereby improving both types of strengths.
- “n” was dissimilar to the activators of the binary mixtures with the best values of two types of strengths, but “Ms” was the same. The binary mixtures with the best compressive values were tested while “n” was set to 6. The best flexural value was obtained while “n” was set to 4.
- In the binary mixtures with MW of 50%, the rising trend of the flexural value between the early and late strengths was similar. However, in the 2BFS-2MW (F) specimens, the rising trend of the compressive value between the early and late strengths was greater when compared to that of the 2BFS-2MW (C) specimens.
- In the 2BFS-2MW (F) specimens, the drying shrinkage after 4 months was smaller when compared to the BFS* reference samples, but that of the 2BFS-2MW (C) specimens was better. The 2BFS-2MW (F) specimens that had a lower “n” value maintained their longitudinal uniformity in a better way when compared to the 2BFS-2MW (C) specimens that had high “n” values.
- In the binary mixtures, a C-S-H bond was obtained with both FTIR and microstructural tests for the BFS* reference samples. However, through XRD, a calcite peak that overlapped with the identical 2θ degree, indicating the C-S-H bond, was observed. Despite the fact that the consistency of 2θ peaks of the binary mixtures was better when compared to the BFS* reference samples, their strengths were smaller. Considering the binary mixtures, such a result showed a role of calcite over the peaks for the identical 2θ degree. Thus, MW and BFS were not in an entire reaction for the activation.

In conclusion, this study attempted to convert two distinct industrial by-products/waste (BFS and MW) into a value-added product through a low-carbon-footprint production process. The binder type developed herein exhibited characteristics suitable for application as a novel binder type, offering favorable workability, strength, and microstructure properties. The current study contributed to an introduction to sustainable binders by facilitating the reutilization of waste materials, by mitigating harmful gas emissions, and adopting a production approach with minimal energy consumption.

## Figures and Tables

**Table 1:** Chemical details of BFS and MW.

Oxides	SiO_2_	Al_2_O_3_	CaO	Fe_2_O_3_	MgO	TiO_2_	CaCO_3_	LOI
**BFS (Wt.%)**	42.36	11.07	31.16	0.79	7.82	2.34	-	-
**MW (Wt.%)**	1.75	0.56	-	0.29	-	0.001	96.82	-
				**BFS**			**MW**	
**Specific Surface Area (m^2^/g)**				0.93			0.26	
**Specific Gravity (g/cm^3^)**				2.72			2.43	

**Table 2:** Experimental parameters of binary mixing samples.

Type of Mix	Type of Activator	Total Na_2_O/Activator (n) (%w)	SiO_2_/Na_2_O (M_s_)	w/b	Cure Conditions ^1^	Additive Material
BFS*-MW	Sodium Silicate and Sodium Hydroxide	2–4–6	1.2–1.5–1.8	0.40–0.45	Steam Oven	Lime (4%)

* %100 BFS paste sample was a control specimen. ^1^ 65 °C for 4 h.

**Table 3:** Parameters for the chosen specimens with the highest values of the binary mixtures.

Mixture Parameters	Chosen Specimen	
	Flexural Strength	Compressive Strength
BFS-MW Ratio (%)	50–50	50–50
“n”	4	6
“Ms”	1.2	1.2
“w/b”	0.4	0.4
Type of Curing	Steam	Steam
Code of Sample	2BFS-2MW/4/1.2/0.4 (F)	2BFS-2MW/6/1.2/0.4 (C)
Reference Sample	BFS*/4/1.5/0.4 (F)	BFS*/6/1.5/0.4 (C)

**Table 4:** FT-IR outcomes for binary mixtures.

	Binary Mixtures			
	Band No	Wavenumber cm^−1^	Attribution	Reference
**2BFS-2MW (F)**	1	3553	O-H Stretching	[38,39]
	2	1637	H-O-H Bending	[38,39]
	3	1412	ν3[CO_3_]^−2^	[38,43]
	4	954	Si-O asymmetric stretching/C-S-H	[38,43,44]
	5	872	C=O/CO_3_^−2^	[25,45,46]
	6	711	C=O/CO_3_^−2^	[31]
**2BFS-2MW (C)**	1	3555	O-H Stretching	[38,39]
	2	1635	H-O-H Bending	[38,39]
	3	1411	ν3[CO_3_]^−2^	[38,43]
	4	954	Si-O asymmetric stretching/C-S-H	[38,43,44]
	5	872	C=O/CO_3_^−2^	[25,45,46]
	6	711	C=O/CO_3_^−2^	[31]

## Data Availability

Data are presented within the article; further inquiries can be directed to the corresponding author.
